# Molecular Docking Characterization of a Four-Domain Segment of Human Fibronectin Encompassing the RGD Loop with Hydroxyapatite

**DOI:** 10.3390/molecules19010149

**Published:** 2013-12-23

**Authors:** Tailin Guo, Wenyuan Kang, Dongqin Xiao, Rongquan Duan, Wei Zhi, Jie Weng

**Affiliations:** 1 School of Life Science and Engineering, Southwest Jiaotong University, Chengdu 610031, China; 2 Key Laboratory of Advanced Technologies of Material, Minister of Education, School of Materials Science and Engineering, Southwest Jiaotong University, Chengdu 610031, China

**Keywords:** fibronectin, hydroxyapatite, molecular docking, RGD loop

## Abstract

Fibronectin adsorption on biomaterial surfaces plays a key role in the biocompatibility of biomedical implants. In the current study, the adsorption behavior of the 7–10th type III modules of fibronectin (FN-III_7–10_) in the presence of hydroxyapatite (HAP) was systematically investigated by using molecular docking approach. It was revealed that the FN-III_10_ is the most important module among FN-III_7–10_ in promoting fibronectin binding to HAP by optimizing the interaction energy; the arginine residues were observed to directly interact with the hydroxyl group of HAP through electrostatic forces and hydrogen bonding. Moreover, it was found that the HAP-binding sites on FN-III_10_ are mainly located at the RGD loop region, which does not affect the interaction between the fibronectin protein and its cognate receptors on the cell surface.

## 1. Introduction

Fibronectin (FN) is a prominent component of extracellular matrices (ECM) and is present at high concentrations (~300 mg/mL) in plasma. It is composed of three types of repeating modules, termed type I, II and III repeats, which are organized into functional domains [1,2,3]. FN mediates its biological effects through binding to the hetero-dimeric transmembrane glycoproteins, integrins, which physically couple the cytoskeleton to the ECM [4]. A majority of integrin-mediated interactions of FN with cells occur through the cell binding triplet Arg-Gly-Asp (RGD loop). Disruption of the FN gene results in an embryonic lethal phenotype, confirming the importance of FN in the cellular development [5] and synthetic RGD loop inhibits cell adhesion on FN coated substrates [6], confirming the importance of RGD loop in the function of FN.

Hydroxyapatite (HAP, [Ca_10_(PO_4_)(OH)_2_]), which is the most abundant apatite in human bone and often considered as “the golden standard” in orthopedics [7], exhibits a desirable bone-tissue response as compared to bare metal implants, including absence of intervening fibrous tissue between bone and implant, lack of inflammation, and strong binding to bone [8]. However, the detailed mechanism underlying this biocompatibility is still not fully understood. The biocompatibility of an implant is related to how the adhering cells interact with the implant surface when the implant is inserted into the body [9]. These cellular responses are in turn influenced by proteins adsorbing on the implant from the body fluids. Accordingly, the arriving cells sense the protein layer covering the surface when they arrive on that surface, thereby “seeing” the implant surface properties through the protein layer [10]. The cellular response therefore depends on the detailed properties of the resulting interfacial protein layer, among which FN is the key one that not only provides a substrate for cell anchorage, but also serves as a regulatory protein in processes such as cell adhesion, motility and proliferation [11,12,13,14,15].

Numerous experimental methods have been developed to investigate the protein adsorption with HA, and researchers have studied the adsorption of proteins on the surface of biomaterials by the methods such as atomic force microscopy (AFM) [16], flow microcalorimetry (FMC) [17], solid state NMR [18], 2D electrophoresis [19], and steered molecular dynamics (SMD) simulations [20]. In this work, the interaction mechanism of FN-III_7–10_, which contains the RGD loop, with HAP molecules was investigated systematically by using a molecular docking strategy. All the binding sites and the binding energy were studied to explore the structural basis and energetic properties of the interactions between FN-III_7–10_ and HAP. Moreover, the binding sites in the RGD loop region of FNIII_10_ and the influence of FNIII_10_ on the binding of other modules to HAP were also characterized in detail for its great importance in promoting cell adsorption.

## 2. Results and Discussion

### 2.1. Identification of Potential HAP-Binding Sites on FN-III_7–10_ Surface and Molecular Docking of HAP to FN

**Figure 1:**
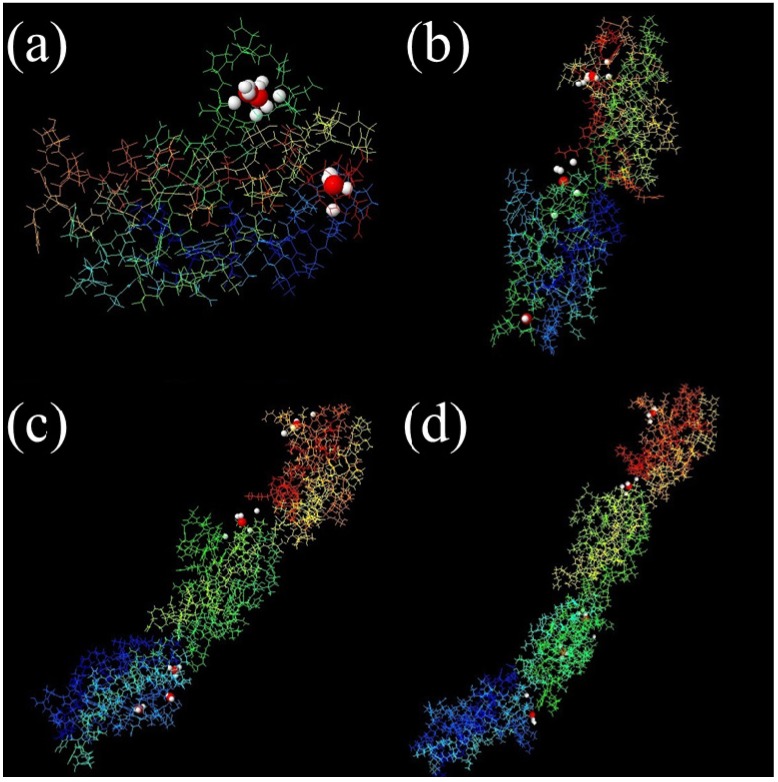
The real ligand (red, hydroxyapatite) binding site and the identified sites on different modules of FN-III_7–10_ (PDB ID: 1PNF). The pocket sites (white) of LIGSITECS, PASS, SURFNET, Q-SiteFinder, Fpocket, ConCavity, GHECOM and POCASA are all from their top 1 predictions and are located in the same cavity where ligand binds. The meta-Pocket site from MPK2 is shown in red sphere. (**a**) FN-III_10_, (**b**) FN-III_9–10_, (**c**) FN-III_8–10_ and (**d**) FN-III_7–10_.

The protein surface can form pockets that are potential binding sites of small-molecule ligands. Therefore, the identification of pocket sites on the protein surface is often the starting point for protein function annotation and structure-based analysis [21]. Also, proper ligand-binding site detection is a prerequisite for protein–ligand docking. Over the past decades, many computational methods have been developed to predict protein–ligand binding sites based on detection of cavities on protein surface. Here, MPK2 was employed to predict the pockets in different fragments of FN, and the results are shown in Figure 1. The predicted pockets are consistent by different methods at the same fragments, and most high scorning pockets exist in the FN-III_10_ fragment and the hinge areas of different modules of FN.

**Figure 2:**
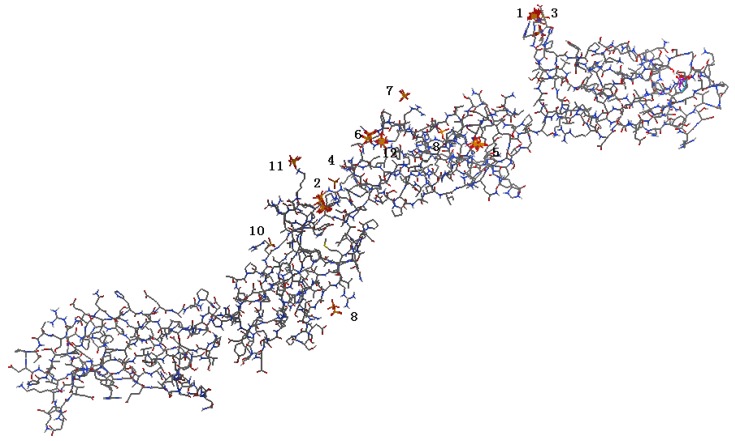
Binding sites on FN surface predicted by AutoDock 4.

**Figure 3:**
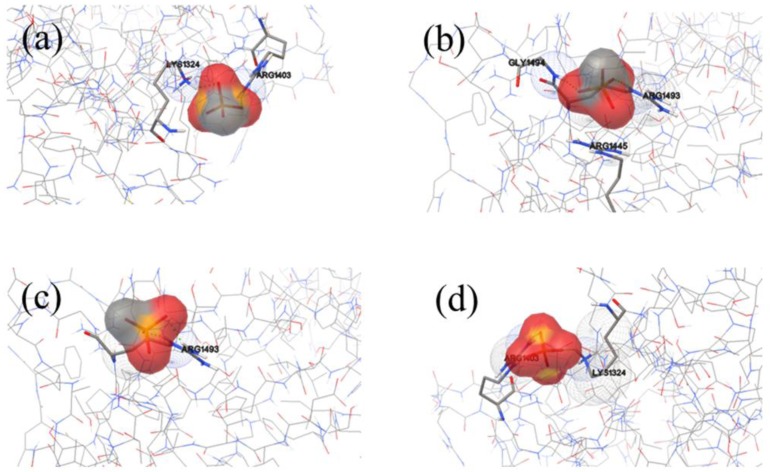
Highest scoring binding sites for top 4 binding clusters between FN (stick) and HAP (ball). The bold sticks show the direct bonding amino acids.

The binding sites and interaction free energies between the FN-III_7–10_ and HAP were further examined using the tool suite of AutoDock 4 [19]. Both the ligand and the receptor were treated as rigid and we only explored the six degrees of translational and rotational freedom, hence excluding any kind of flexibility. There were multiple binding sites detected at every binding cluster and ten sites of minimum binding energy were selected. The resulting binding sites are shown in Figure 2. A total of 13 predicted binding clusters in FN-III_7–10_ were detected, which separately locate at the FN-III_8_, FN-III_9_, FN-III_10_ and the hinge region. While the sites with the lowest binding energy were in FN-III_10_, and there were no sites found in FN-III_7_. All the results were consistent with the results predicted by MPK2. For each binding cluster, the sites of the lowest binding energy were further investigated with respect to their interacting amino acids. The residues Arg1493, Arg1445, Gly1494, Lys1324, Arg1403, Arg1371, Phe1366, Ser1367 and Gly1368 were from cluster 1–4 (Figure 3), which formed the lowest binding energy sites in all clusters. Arginine is the most important amino acid in the binding of HAP to FN with the highest frequency of occurrence. The side chain of arginine consists of a 3-carbon aliphatic straight chain, and the distal end of which is capped by a complex guanidinium group. With a p*K*_a_ of 12.48, the guanidinium group is positively charged in neutral, acidic and even most basic environments, and thus imparts basic chemical properties to arginine. Because of the conjugation between the double bond and the lone pairs of nitrogen atoms, the positive charge is delocalized, enabling the formation of multiple hydrogen bonding, which prompts the binding with the HAP entity (rich of hydroxyl groups).

**Figure 4:**
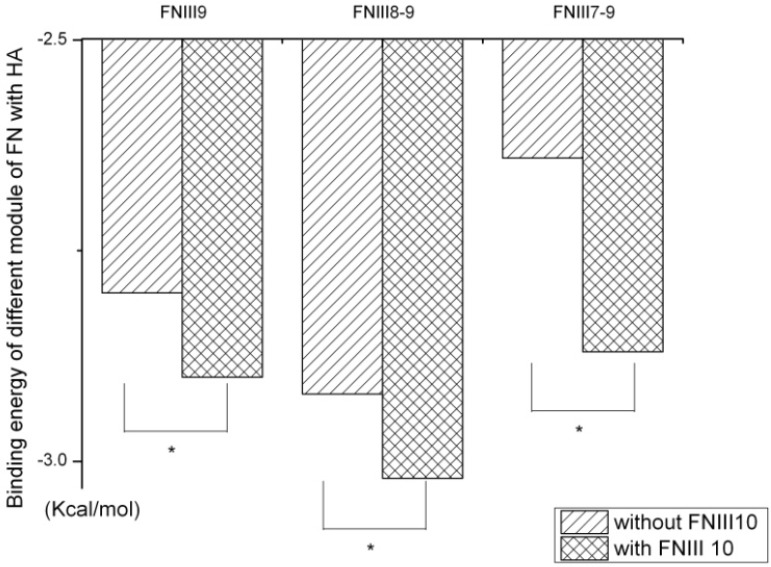
The lowest binding energy between different modules of FN-III_7–10_ and HAP nanoparticle. Without FNIII_10_, the binding energy between other different modules of FNIII_7–9_ and their combinations will be reduced significantly.

FN-III_7–10_ consists of four module domains and each is relatively independent. All modules and their different combinations were also analyzed with AutoDock, which suggested that FN-III_10_ is the most important one in the interaction with HAP, which can efficiently improve the interaction preference between the FN and HAP by optimizing their binding free energy (Figure 4). When combined with FN-III_10_, the lowest binding energy of FN-III_9_, FN-III_8–9_ and FN-III_7–9_ were decreased dramatically. In fact, short peptides containing the sequence motif Arg-Gly-Asp (RGD) from FN-III_10_ will bind to integrins themselves additional residues in FN-III_9_, the so-called synergy region, have also been implicated in this interaction [3]. Thus, it is evident that mutual promotion is a common phenomenon in this system. The FN-III_7_, FN-III_8_ and FN-III_9_, however, are not able to enhance interaction affinity of HAP with other modules of the FN.

### 2.2. Analysis of HAP-Binding Site on RGD Loop

FN binding to the integrin receptors on most cells is modulated by interactions with a loop containing a RGD motif in the tenth FN-III module. Approximately one-third of the integrin receptor family recognizes RGD. The tripeptide itself improves the resistance of isolated islets against apoptosis [22]. A distinct sequence in the ninth FN-III module (PHSRN in hFN-III_9_ and PPSRN in mFN-III_9_), the synergy region, is also implicated in the cell attachment interaction [23,24]. The cell-binding RGD loop itself is well-ordered, and extends 1 nm from the core of the molecule [25]. Therefore, whether the binding of HAP to FN-III_10_ can interfere in the interaction between the RGD and integrin will be important for the biocompatibility of HAP.

**Figure 5:**
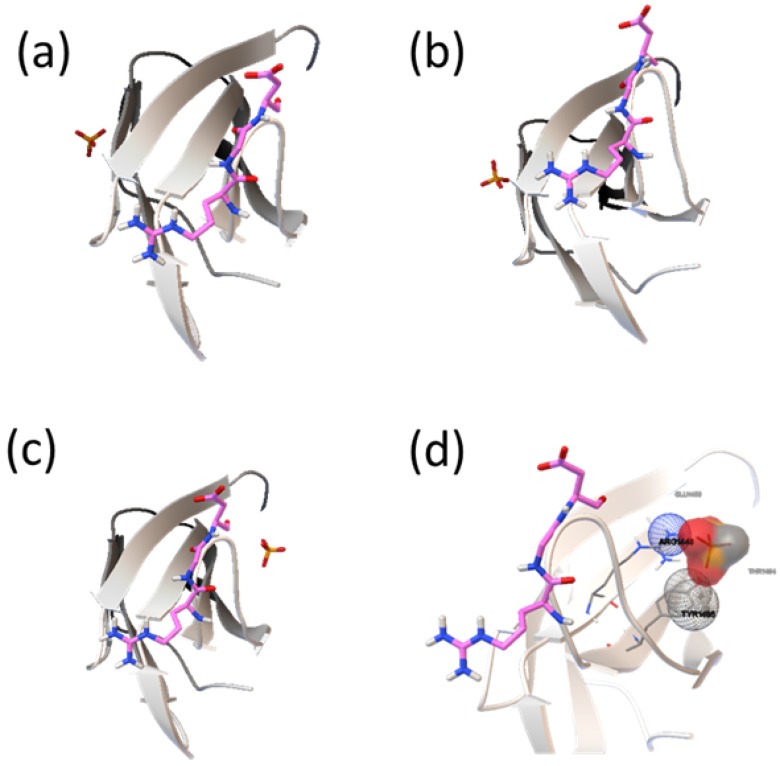
Binding model of FNIII_10_ (carton model) and HAP molecular (stick) near RGD loop (stick model). (**a**) cluster 1, (**b**) cluster 2, (**c**) cluster 3 and (**d**) further analysis about cluster 3 for the HAP binding sites near the RGD loop.

Much attention has been paid on the binding sites of HAP on FN-III_10_ surface (Figure 5), and the amino acids involved in the interaction. Among all the binding sites, there are the lowest binding energy and most sites in cluster 1, where HAP is at the sides of RGD loop and the contacting residues are Ala1472, Lys1469 and Thr1473. The binding sites on cluster 2 are also at the sides of RGD loop, and the residues are Val1465, Lys1469 and Pro1466. Although binding sites in cluster 3 are nearby the RGD loop, but the interaction energy is higher, and the binding probability is the lowest. In a view, most the binding sites of HAP on FN-III are existed in the side region of RGD loop and does not directly interact with the residues in the loop (Arg1493, Gly1494 and Asp1495) to interfere the function of RGD in cell adhesion.

## 3. Experimental

### 3.1. Setup of FN-III_7–10_ and HAP Structures

**Figure 6:**
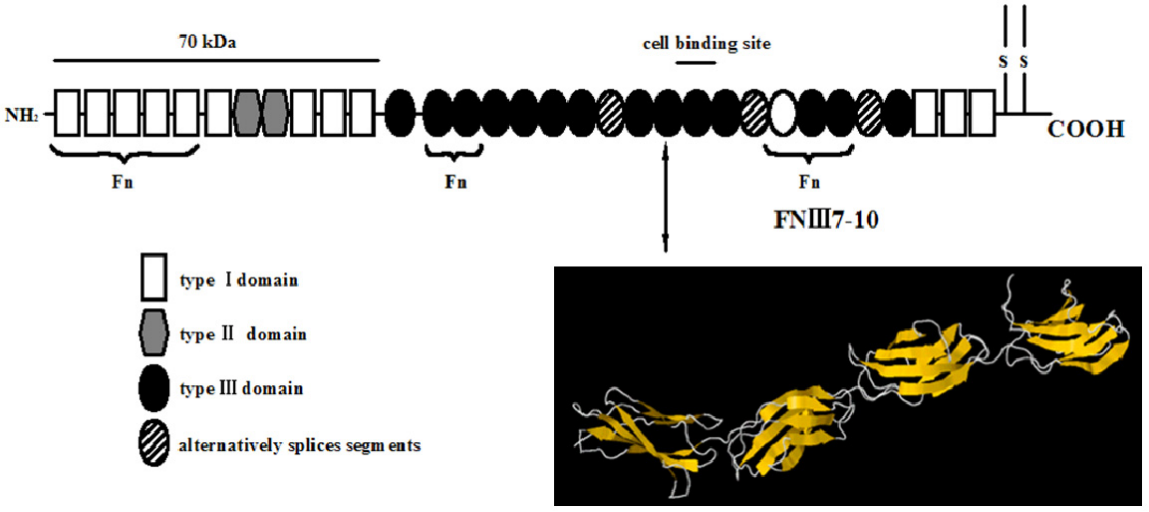
Schematic representation of fibronectin modules and structures [20]. Fibronectin is a 450–500 kd dimeric protein composed of more than 20 modules per monomer. Two monomeric strands linked by two disulfide bridges and each monomer contains three types of modules, types I–III, and/or alternatively splices of segments that are found inserted or found missing in various spliced forms of fibronectin. Arrow specified the 7–10th type III module of fibronectin (FN-III7-10), which contains cell recognition site.

**Figure 7:**
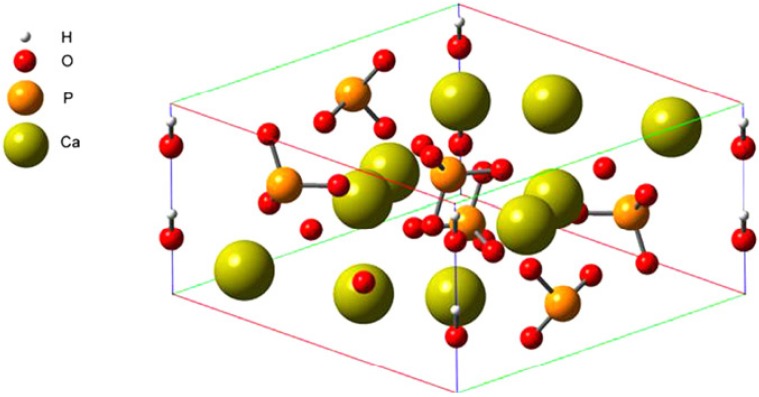
Schematic representation of a HAP unit cell [30].

The high-resolution crystal structure (Figure 6) of FN-III_7–10_ was retrieved from the Protein Data Bank database with the access ID: 1FNF. The cell structure of HA [Ca_9_Na_0.5_(PO_4_)_4.5_(CO_3_)_1.5_(OH)_2_] (Figure 7) can easily accommodate a great variety of substitutes, including both anionic and cationic [26,27]. The compact arrangement of PO_4_ groups in the structure provides two kinds of channels containing calcium ions [28]. Four per unit cell among them, called Ca I, are in columns parallel to the *c*-axis and surrounded by nine oxygen atoms [three O(1), three O(2) and three O(3)]. The others, six per unit cell, called Ca II, are surrounded by one O(1), one O(2), four O(3) and one OH ion. They form equilateral triangles centered at the unit-cell corners. The large size of the Ca II triangle allows motion of the hydroxyl ion along the column axis [29].

### 3.2. The FN-III_7–10_ Binding Site Prediction with MPK2

Meta Pocket 2.0 (MPK2) is a consensus method in which the pocket sites predicted by eight methods—LIGSITECS, PASS, Q-Site Finder, SURFNET, Fpocket, GHECOM, Con-Cavity and POCASA—are combined to improve the prediction success rate. There are three steps in the MPK2 procedure: calling-based methods, generating meta-pocket sites and mapping ligand-binding residues [31]. Different PDB files (FN-III_10_, FN-III_9–10_, FN-III_8–10_ and FN-III_7–10_) were submitted one-by-one to the MPK2 web server [32] to perform an automatic site search. Then, only the top three pocket sites in each method are taken into further consideration. Therefore, we have a total of 24 pocket sites for each protein, which are clustered using a simple hierarchical clustering algorithm, according to their spatial similarity (distance based).

### 3.3. Performing Molecular Docking with AutoDock 4

Molecular docking is a computational method that predicts the binding of a ligand to a receptor [33]. Hence, it is an important tool in studying receptor–ligand interactions [34]. AutoDock is the most popular docking programs, which uses a Lamarckian genetic algorithm (LGA), but encompasses also a Monte Carlo simulated annealing and a traditional genetic algorithm, to position ligand binding modes within the active pocket of protein receptor. Simulated annealing issued for searching conformations, allowing several tensional degrees of freedom in a flexible ligand to be searched during the docking experiment, but with the limitation that it may not always find the global minimum conformation. A grid-based technique is used for energy evaluation at each step of the simulation, providing a detailed energetic model at reasonable computational cost. All the courses were done according to the protocol of AutoDock 4 [35].

## 4. Conclusions

FN-III10 is the most important module among FN-III7–10 in promoting fibronectin binding to HAP by optimizing the interaction energy; the arginine residues were observed to directly interact with the hydroxyl group of HAP through electrostatic forces and hydrogen bonding. The results verify that the HAP-binding sites on FN-III10 are mainly located at the RGD loop region, which does not affect the interaction between the fibronectin protein and its cognate receptors on the cell surface.
